# Supplementary material for: Masculinity norms and occupational role orientations in men treated for depression
Source: PLoS One. 2020 May 26;15(5):e0233764. doi: 10.1371/journal.pone.0233764 (PMC7250462; doi:10.1371/journal.pone.0233764)
Supplement: S1 Fig — Means and 95% confidence intervals adjusted for age, partner, education, unemployment, income, duration of illness, duration of untreated illness (only in the DSS models), recruitment setting. (DOCX) [file pone.0233764.s001.docx]

Figure S1: Depression Stigma Scale (DSS) subscales and duration of untreated illness (DUI) by latent class assignment. Means and 95% confidence intervals adjusted for age, partner, education, unemployment, income, duration of illness, duration of untreated illness (only in the DSS models), recruitment setting.
