# Supplementary material for: Masculinity norms and occupational role orientations in men treated for depression
Source: PLoS One. 2020 May 26;15(5):e0233764. doi: 10.1371/journal.pone.0233764 (PMC7250462; doi:10.1371/journal.pone.0233764)
Supplement: S1 Table — (DOCX) [file pone.0233764.s002.docx]

| **Table S1: Cronbach’s alpha of the study instruments in the current sample** | | |
| --- | --- | --- |
| **Instruments and subscales** | **No of Items** | **Cronbachs alpha** |
| **MRNS** anti-femininity | 7 | 0.76 |
| **MRNS** toughness | 8 | 0.71 |
| **MRNS** status | 11 | 0.84 |
| **AVEM** priority of work | 6 | 0.89 |
| **AVEM** professional ambition | 6 | 0.89 |
| **AVEM** over-commitment | 6 | 0.87 |
| **AVEM** perfectionism | 6 | 0.87 |
| **AVEM** distancing ability | 6 | 0.90 |
| **AVEM** resignation | 6 | 0.88 |
| **AVEM** offensive coping | 6 | 0.79 |
| **AVEM** calmness | 6 | 0.77 |
| **AVEM** experience of success | 6 | 0.92 |
| **AVEM** life satisfaction | 6 | 0.84 |
| **AVEM** social support | 6 | 0.78 |
| **PHQ-9** Depression | 9 | 0.87 |
| **PHQ-7** Anxiety | 7 | 0.90 |
| **PHQ-15** Somatic symptoms | 14 | 0.83 |
| **PHQ-SADS** total | 30 | 0.94 |
