# Supplementary material for: Masculinity norms and occupational role orientations in men treated for depression
Source: PLoS One. 2020 May 26;15(5):e0233764. doi: 10.1371/journal.pone.0233764 (PMC7250462; doi:10.1371/journal.pone.0233764)
Supplement: S2 Table — (DOCX) [file pone.0233764.s003.docx]

| **Table S2: Means and standard deviations of the dimensions of the Male Role Norms Scale (MRNS) and the Work Related Behavior and Experience Scale (AVEM) by latent class assignment** | | | | |
| --- | --- | --- | --- | --- |
| **N (%)** | **Class 1**  85 (34.0) | **Class 2**  58 (23.2) | **Class 3**  107 (42.8) | **sig. of mean differences^1)^**  p <=0.05 |
| **MRNS** anti-femininity | 14.4 (4.5) | 18.5 (5.4) | 13.5 (3.9) | 1,3 < 2 |
| **MRNS** toughness | 19.7 (4.6) | 24.2 (4.9) | 19.1 (4.2) | 1,3 < 2 |
| **MRNS** status | 29.3 (6.3) | 38.9 (6.6) | 29.8 (6.9) | 1,3 < 2 |
| **AVEM** priority of work | 11.9 (4.3) | 20.4 (4.6) | 15.0 (5.1) | 1 < 3 < 2 |
| **AVEM** professional ambition | 11.8 (3.6) | 21.5 (4.6) | 17.4 (4.8) | 1 < 3 < 2 |
| **AVEM** over-commitment | 16.0 (5.2) | 24.8 (3.4) | 18.7 (4.5) | 1 < 3 < 2 |
| **AVEM** perfectionism | 20.2 (5.1) | 25.8 (3.5) | 22.4 (4.3) | 1 < 3 < 2 |
| **AVEM** distancing ability | 15.9 (5.3) | 9.9 (3.6) | 16.6 (5.3) | 1,3 > 2 |
| **AVEM** resignation | 22.5 (4.8) | 24.1 (3.6) | 17.7 (3.8) | 1,2 > 3 |
| **AVEM** offensive coping | 14.1 (3.2) | 17.3 (3.9) | 19.3 (3.1) | 1 < 2 < 3 |
| **AVEM** calmness | 15.6 (3.8) | 13.4 (4.0) | 18.2 (3.7) | 3 > 1 > 2 |
| **AVEM** experience of success | 14.0 (5.4) | 17.5 (5.9) | 21.2 (4.2) | 1 < 2 < 3 |
| **AVEM** life satisfaction | 11.2 (3.3) | 12.3 (3.1) | 18.2 (3.5) | 1,2 < 3 |
| **AVEM** social support | 17.5 (4.6) | 17.0 (3.1) | 22.2 (3.7) | 1,2 < 3 |
| 1) oneway ANOVA; Scheffe test for post hoc comparisons | | | | |
