# Supplementary material for: Masculinity norms and occupational role orientations in men treated for depression
Source: PLoS One. 2020 May 26;15(5):e0233764. doi: 10.1371/journal.pone.0233764 (PMC7250462; doi:10.1371/journal.pone.0233764)
Supplement: S3 Table — (DOCX) [file pone.0233764.s004.docx]

| **Table S3: Raw mean differences of PHQ-SADS subscales, DSS subscales and DUI by latent class assignment** | | | | |
| --- | --- | --- | --- | --- |
| **Latent class assignment** n (%) | **Class 1**  85 (34.0) | **Class 2**  58 (23.2) | **Class 3**  107 (42.8) | **mean**  **differences**  p < 0.05**^1)^** |
| **Depressive Symptoms PHQ-9** mean (SD) | 16.1 (6.2) | 17.3 (5.1) | 10.9 (6.0) | 1, 2 > 3 |
| **Anxiety Symptoms (PHQ-7)** mean (SD) | 12.4 (5.1) | 14.2 (5.0) | 8.8 (5.2) | 1, 2 > 3 |
| **Somatization Symptoms (PHQ-15) ^2)^** mean (SD) | 12.3 (5.1) | 13.2 (5.8) | 9.1 (5.2) | 1, 2 > 3 |
| **Depression Stigma Scale (DSS) Personal Stigma** mean (SD) | 22.8 (6.7) | 26.9 (7.2) | 20.5 (5.3) | 2 > 1 > 3 |
| **Depression Stigma Scale (DSS) Perceived Stigma** mean (SD) | 39.3 (6.8) | 41.4 (8.8) | 35.3 (7.5) | 2 > 1 > 3 |
| **Duration of Untreated Illness (DUI) mean (SD)** | 3.3 (5.8) | 5.7 (8.7) | 3.0 (5.3) | 2 > 3 |
| 1) oneway ANOVA; Scheffé test for post hoc comparisons  2) One item asking for menstruation complains was removed form the scale | | | | |
