# Supplementary material for: Masculinity norms and occupational role orientations in men treated for depression
Source: PLoS One. 2020 May 26;15(5):e0233764. doi: 10.1371/journal.pone.0233764 (PMC7250462; doi:10.1371/journal.pone.0233764)
Supplement: Attachment — Submitted filename: response to reviewers.docx [file pone.0233764.s008.docx]

Reviewers' comments:

Reviewer's Responses to Questions

Comments to the Author

1. Is the manuscript technically sound, and do the data support the conclusions?

The manuscript must describe a technically sound piece of scientific research with data that supports the conclusions. Experiments must have been conducted rigorously, with appropriate controls, replication, and sample sizes. The conclusions must be drawn appropriately based on the data presented.

Reviewer #1: Partly

Reviewer #2: Partly

________________________________________

2. Has the statistical analysis been performed appropriately and rigorously?

Reviewer #1: I Don't Know

Reviewer #2: I Don't Know

________________________________________

3. Have the authors made all data underlying the findings in their manuscript fully available?

The PLOS Data policy requires authors to make all data underlying the findings described in their manuscript fully available without restriction, with rare exception (please refer to the Data Availability Statement in the manuscript PDF file). The data should be provided as part of the manuscript or its supporting information, or deposited to a public repository. For example, in addition to summary statistics, the data points behind means, medians and variance measures should be available. If there are restrictions on publicly sharing data—e.g. participant privacy or use of data from a third party—those must be specified.

Reviewer #1: No

Reviewer #2: No

________________________________________

4. Is the manuscript presented in an intelligible fashion and written in standard English?

PLOS ONE does not copyedit accepted manuscripts, so the language in submitted articles must be clear, correct, and unambiguous. Any typographical or grammatical errors should be corrected at revision, so please note any specific errors here.

Reviewer #1: Yes

Reviewer #2: Yes

________________________________________

5. Review Comments to the Author

Please use the space provided to explain your answers to the questions above. You may also include additional comments for the author, including concerns about dual publication, research ethics, or publication ethics. (Please upload your review as an attachment if it exceeds 20,000 characters)

Reviewer #1: Introduction

The question of the role of masculinity in delaying treatment seeking is an important one, however the study as presented does not focus sufficiently on this. In both the introduction and the discussion the focus is much more on male role orientations and work roles in relation to depression. The problem with that focus is that the sample are all in treatment for depression and there is no non-depressed comparison group. Thus, all the study can really examine is different configurations of masculinity that can be discerned among depressed men. The authors need to make a much stronger case about why this warrants attention, and re-title the paper to reflect that focus. I believe the question of masculinity and delay in help-seeking, and the role of stigma in that, is of much more interest and if the paper could be refocused around that it would be of more value.

*We agree with the reviewer that the main focus of the manuscript is the association between masculinity and work-role orientation with psychiatric symptoms, stigmatization and the delay of treatment in men already treated for depression. In our view the investigation of this question is important because it can improve our understanding of how relevant aspects of masculinity are in the treatment process.*

*We changed the title of the manuscript to make our intention more clear and we tried to focus the introduction.*

The inclusion of ‘burnout’ is at no point explained – is it a proxy for poor mental health? Or a condition in its own right? Or a recruiting convenience? It needs to be discussed in the introduction and a rational provided for combining a burnout sample with a depressed sample.

*The main reason for including people with burnout was that we suspected that particularly by family doctors the diagnosis of burnout will often be used when they guess that patients refuse to accept the diagnosis of depression. We therefore expected that by including patients treated for burnout we would get access to patients who are reluctant to accept a diagnosis of a mental disorder. However, due to the fact that only very few patients (n = 15) treated for burnout participated in the study, we decided to exclude these patients from our analyses to get a more homogenous sample. We re-calculated all statistical analyses on the basis of the new sample.*

Methods.

The paper doesn’t report on the qualitative element of the study so I don’t think it necessary to present the paper as based on a mixed-methods design.

*The study was conceptualized as a mixed-method study in the sense that the results of the standardized quantitative part of the investigation should be used as the basis for the selection of study participants for further qualitative inquiries. However, we agree with the reviewer that the current manuscript reports only results of the standardized quantitative part of the study. Therefore, we followed the recommendation not denoting the study as mixed-method in this manuscript.*

Can the cities or towns where the study took place be named in addition to the country and the years of the study.

*According to the recommendation of reviewer 1 we named the main cities and described the study region more detailed in the method section.*

The inclusion of ‘burnout’ as an inclusion criteria needs much more explanation – i.e. what is ‘burnout’, how is it diagnosed, what is the relationship between burnout and depression – basically we need sufficient information to be able to judge that combining these two samples provides a suitable sample for the analysis undertaken and the conclusions drawn.

*See above.*

We need more information on participants recruited through newspaper advertisements – how were they screened.

*We described the recruitment process more detailed and we present an extra table with the description of sample characteristics broken down by recruitment setting.*

We need more information on the format of data collection – was it self-complete questionnaires, was it by face-to-face interview? Was it conducted by clinic staff or by the researchers etc.

*We described the data collection more detailed in the method section.*

Delay in psychiatric treatment – notes that delay is calculated based on “the year in which a psychiatric treatment for depressive symptoms was used for the first time”. In table 1 delay is reported as ‘months untreated’ – need to explain how those months were calculated i.e. from January in the year treatment was initiated?

*We asked patient in which year they had depressive symptoms for the first time and in which year they had their first professional treatment contact and computed the delay of treatment by calculating the difference between both years. We agree with the evaluator that the use of months as a unit of treatment delay suggests a higher measurement accuracy than was actually implemented. We therefore describe the assessment of the variable more detailed and changed the unit of measurement into years.*

Also, if receiving treatment for burnout is an eligibility criterion is it possible that some of the men might never have been treated for depression?

**See above.**

Overall, we need more clarity around the diagnostic status of the sample at recruitment. This also needs to be reported in Table 1.

*We reported the clinical status of the study participants by means of the PHQ-9 cut of values for the severity of depression in table 1.*

Results.

It would be good to see descriptive statistics for the various measures in Table 1 as well. And as mentioned above, diagnostic status (at minimum if they are being treated for depression or for burnout’.

*In addition to the PHQ-9 cut-off values we reported the means and standard deviations of all PHQ subscales broken down by latent-class in table 2.*

The text describing figure 5 appears to have errors – the second sentence compares class 2 to class 2. There are also some spelling errors in that paragraph.

*We are sorry for this error and corrected the formulation.*

Discussion

The discussion would benefit from being more focused on the question that the paper describes in the title and abstract – the relationship between masculine norms and work role and the delaying of treatment seeking, and the putative role of stigma in that relationship. The focus is predominantly on differences in adherence to traditional masculinity and occupation role orientations in a depressed/burnout sample, and the authors have not made a strong argument about why this is important to study.

*We agree that our study is mainly focused on the relationship of masculinity and work-role orientations with the psychological well-being of depressed patients. We therefore tried to underline the clinical relevance of these associations for depression therapy in the introduction and in the discussion.*

Much of the discussion then describes the relationship of masculinity and work role orientation which is not relevant to the original research question. Moreover the assumption made “that traditional masculine role orientation is not harmful per se to mental health, but depends on its functional and contextual meaning in the work environment” cannot be supported by the study as the sample is comprised of only men with depression.

***We agree that our study design and the study sample does not allow general conclusions about the effects of masculinity norms and work-role orientations on the etiology of depressive symptoms and we therefore focused our conclusion to the population of men with depression which is represented by our sample***

Reviewer #2: Overall, the paper paper is focused on an important gap in the literature and makes a contribution to the field. However, there were some limitations which must be seriously taken into account. These include generalizability of the findings without more information about recruitment process and sample, types of occupations of the participants, as well as the recall bias that may be affecting one of the main outcomes (delay in treatment). The study can only provide information on men who ultimately seek care, and there may be a much wider pool of men who never seek treatment at all, who may not be represented in these results and this should be acknowledged.

We addressed the limitation of generalizability in the limitation section.

There is some convoluted writing and significant typos. It may be useful to have the paper copy edited (perhaps professionally) for standard English when revising the paper.

*We have completely revised the language of the manuscript, including professional editing*

Abstract:

• More detail is needed on the methods. How were patients recruited? Convenience sample? What measures were used? Are they validated measures? Was the study quantitative or qualitative? What was the participation rate? Some of this information can be provided solely in the manuscript, but more detail is needed in the abstract as well.

*We described the study methods in the abstract more detailed.*

• Last sentence of the conclusions should read: “in the development of strategies to reduce the delay…and to tailor the psychiatric treatment…”

*We thank the reviewer for this advice and made the recommended changes.*

Introduction:

• The introduction needs to be more focused on the importance of the work environment earlier. As the introduction currently reads, it is not clear immediately that the focus will be occupational ‘identity’ or norms.

*We reformulated the introduction to make the focus on masculinity and work-role orientations more clear.*

• Page 3, last sentence of the first paragraph: “negatively associated with depressive symptoms” is unclear. Can say, “was associated with lower depressive symptoms,” or other language to make the association clearer.

*We thank the reviewer for this advice and made the recommended changes.*

• Page 4, first sentence: Please specify what the ‘control populations’ were. Individuals without depression?

*We added the missing information about the control population.*

Methods:

• The fact that this is a mixed-methods study needs to be in the abstract.

*Since the manuscript provides only results from the quantitative part of the study, we followed the recommendation of reviewer 1 and deleted the note on the mixed-method character of the study from the manuscript.*

• Please specify whether the measures were validated measures from the very beginning (first paragraph of study design section).

*Done.*

• There are multiple typos in the methods section.

*We regret the negligence and have tried to correct all mistakes.*

• When you say that a ‘sub-sample of study participants were selected on the bases if the results’ please specify what the basis of this decision was. Will help the reader understand generalizability of the results. Who was chosen to be interviewed?

*For only quantitative data were used in this manuscript and the qualitative part of the study had no influence on the quantitative results, we followed the recommendation of reviewer 1 and deleted all notes on the qualitative part of the study from this manuscript.*

• Why was this age rage chosen? Please justify the rationale. Was the idea to choose only working-age men? If so, please specify.

*Yes, because our particular interest in work-role orientations we included only men below the usual age of retirement, which in Germany is 65 years.*

• For some readers, such as myself, we are unfamiliar with the term ‘psychosomatic hospitals.’ Please provide a bit more background on these.

*We specified the difference between psychiatric and psychosomatic hospitals in the method section.*

• The paper should specify how many patients came from each of the different settings, as again, this will affect generalizability.

*We presented detailed information about the recruitment in table 1.*

• Please provide more info on the recruitment process. Were patients asked to participate if they were having depressive symptoms, if they were in treatment for depression, etc. What did the recruitment messaging say?

*We presented a more detailed description of the recruitment process and the press invitation in the method section.*

• It says that eligible patients were contacted by doctors or clinical staff about willingness to participate. Was this the case even for participants who came in via the press release?

*We added the information that patients who reported on the press release were directly contacted by the study workers.*

• Cronbach’s alpha numbers are part of results, not methods. Please move these to the results section.

*Cronbach’s Alpha computed with our data are now presented in the result section.*

• Please provide the full name for all acronyms (such as AVEM on page 6).

*Done.*

• Some questions were added to validated scales. This would be unvalidated measures unless validated elsewhere. Please specify if that was the case.

*We computed Cronbach’s Alpha for all measures used in the study. The psychometric properties of the DSS subscales with the additional items were better than those reported in the literature.*

• For ‘delay of treatment,’ which is an important outcome in this study, please provide further information on how this was assessed. There seems to be a lot of room for recall bias, which is inherently problematic and should be acknowledged in the limitations section.

*We agree that recall bias is a problem and we addressed this in the limitation section.*

• The information on the LPA could potentially be included in an appendix if allowed, rather than in the body of the paper.

*We would like to leave the information in the methods section because the use of LPA is not so common in medical science.*

• ‘one-way analyses of variance’= ANOVA and should be specified.

*In order to reduce the amount of results presented, we no longer refer to ANOVA results.*

Results:

• Many of the covariates were not described in the methods. How is ‘higher education’ defined? College degree? More than college degree? Duration of illness is lacking units. Is this years, months, etc. Why was the cut-off of >3000 euros used? Please provide the rationale for this decision.

*We provided more detailed information on covariates in the statistical method section.*

*The 3000 € cut-off was used because it is the average net household income in Germany at the year of the study was about 3.400 € and the mean and the median net household income category in our study were 2000-3000 €*

• Page 13, last sentence: the sentence structure is very convoluted, which is obscuring the meaning of the sentence. Do you mean, ‘participants in latent class 1 were less likely to have a partner…than participants in latent class 3’?

*We have reformulated the sentence*

• Page 15, last paragraph, second sentence: class 2 is compared to class 2.

*We corrected this error*

• The authors seem to be presenting an overwhelming amount of results that are hard to interpret. I would consider trying to figure out what the main results and take-away findings are and moving all others to an appendix section.

*We agree that the presented results are in part redundant. However, as a consequence of our decision to exclude participants who were treated for burnout and not for depression we also decided to present only the results for the PHQ scales and delete the results related to symptoms of burnout.*

• Many typos throughout the results section.

*We did a new language editing of the whole manuscript*

Discussion:

• The first sentence of the discussion section was not well set up in the introduction. It was unclear that this was the hypothesis that the authors were challenging.

*We reformulated the introduction and also the first sentence of the discussion*

• The bullets in the discussion section are more re-hashing of the results and are actually much clearer summaries than the ones provided in the actual results section.

*We regard the summary of the results of the latent profile analysis as useful for helping the reader to follow the discussion.*

• ‘Sex’ and ‘gender’ are used interchangeably throughout the paper, when the authors are in fact referring to ‘gender’ only throughout.

*We changed “sex” into “gender” if appropriate*

• Page 19: there should be something in the discussion about how the info from the study could be used in practice.

*We discussed this point more detailed*

Limitations section:

• Self-selection of participants is an important limitation, as it limits the generalizability of results. Can only speak for patients IN treatment who were willing to participate in the study. Cannot speak for men who do not seek treatment at all or who are unwilling to participate in the study. Generalizability should be listed as a limitation specifically.

• It is also a limitation that delay is all self-report, which is highly subjected to recall bias. Unclear how severity of illness, duration of treatment, etc may impact recall of treatment delay.

*We discussed these limitations in detail in the limitation section*

• A major limitation is that type of occupation is not included in the study results. What type of work did the men included in the study do? Blue collar vs white collar? This seems like it could strongly affect or influence the findings.

*We included blue collar vs. white collar work as a control variable*

Conclusions:

• Can unfortunately not speak about the ‘treatment process,’ only ‘seeking treatment’ or ‘access’ to treatment.

• The last sentence, while true, seems unrelated to the study. More could be said specifically about the impact or use of the findings.

*We reformulated the conclusion to keep closer to the presented results*

________________________________________

6. PLOS authors have the option to publish the peer review history of their article (what does this mean?). If published, this will include your full peer review and any attached files.

If you choose “no”, your identity will remain anonymous but your review may still be made public.

Do you want your identity to be public for this peer review? For information about this choice, including consent withdrawal, please see our Privacy Policy.

Reviewer #1: No

Reviewer #2: No

[NOTE: If reviewer comments were submitted as an attachment file, they will be attached to this email and accessible via the submission site. Please log into your account, locate the manuscript record, and check for the action link "View Attachments". If this link does not appear, there are no attachment files to be viewed.]

While revising your submission, please upload your figure files to the Preflight Analysis and Conversion Engine (PACE) digital diagnostic tool, https://pacev2.apexcovantage.com/. PACE helps ensure that figures meet PLOS requirements. To use PACE, you must first register as a user. Registration is free. Then, login and navigate to the UPLOAD tab, where you will find detailed instructions on how to use the tool. If you encounter any issues or have any questions when using PACE, please email us at figures@plos.org. Please note that Supporting Information files do not need this step.
